# 

**Published:** 1892-04

**Authors:** 


CONTENTS

Vol. 39. No. 4.

| | PAGE. |
|---|---|
| Special to News Dealers | 73 |
| Treatment of La Grippe | 73 |
| Talks with Dr. Mandeville | 76 |
| Functional Exercise | 78 |
| Doctors Disagree | 80 |
| Needed Sleep | 81 |
| Cleanliness | 83 |
| The Old Fashioned Girl | 84 |
| Biting the Finger-nails | 85 |
| The Eyes | 86 |
| Why Men Don't Marry | 88 |
| Learn to Walk | 89 |
| Let the Girls Romp | 90 |
| The Teeth | 91 |
| Hours of Sleep | 91 |
| Physical Sins | 92 |
| Miscellaneous | 92 |
| Literary | 94 |
| Why Do I Love Her | 96 |

INDEX TO ADVERTISEMENTS OF HALF A PAGE AND OVER.

| | PAGE. |
|---|---|
| Woman's Work | I |
| Cornish & Co | II |
| Bovinine | IV |
| Ayer's Cherry Pectoral | V |
| Hall's Journal Premiums | VII |
| Washington Improvement Co. | VIII |
| Magee Emulsion Co | X |
| Humanity and Health | X |
| Royal Baking Powder | XI |
| Buffalo Lithia Water | XI |

